# Horizontally acquired divergent O-antigen contributes to escape from cross-immunity in the classical bordetellae

**DOI:** 10.1186/1471-2148-13-209

**Published:** 2013-09-25

**Authors:** Sara E Hester, Jihye Park, Laura L Goodfield, Heather A Feaga, Andrew Preston, Eric T Harvill

**Affiliations:** 1 Department of Veterinary and Biomedical Sciences, The Pennsylvania State University, W-210 Millennium Science Complex, University Park, PA, 16802, USA; 2 Graduate Program in Biochemistry, Microbiology and Molecular Biology, The Pennsylvania State University, University Park, PA, USA; 3 Graduate Program in Bioinformatics and Genomics, The Pennsylvania State University, University Park, PA, USA; 4 Graduate Program in Immunology and Infectious Disease, The Pennsylvania State University, University Park, PA, USA; 5 Department of Biology and Biochemistry, University of Bath, Bath, UK

**Keywords:** O-antigen, Horizontal gene transfer, Selective advantage, GC content, Recombination, *Bordetella*

## Abstract

**Background:**

Horizontal gene transfer (HGT) allows for rapid spread of genetic material between species, increasing genetic and phenotypic diversity. Although HGT contributes to adaptation and is widespread in many bacteria, others show little HGT. This study builds on previous work to analyze the evolutionary mechanisms contributing to variation within the locus encoding a prominent antigen of the classical *bordetellae*.

**Results:**

We observed amongst classical *bordetellae* discrete regions of the lipopolysaccharide O-antigen locus with higher sequence diversity than the genome average. Regions of this locus had less than 50% sequence similarity, low dN/dS ratios and lower GC content compared to the genome average. Additionally, phylogenetic tree topologies based on genome-wide SNPs were incongruent with those based on genes within these variable regions, suggesting portions of the O-antigen locus may have been horizontally transferred. Furthermore, several predicted recombination breakpoints correspond with the ends of these variable regions. To examine the evolutionary forces that might have selected for this rare example of HGT in bordetellae, we compared *in vitro* and *in vivo* phenotypes associated with different O-antigen types. Antibodies against O1- and O2-serotypes were poorly cross-reactive, and did not efficiently kill or mediate clearance of alternative O-type bacteria, while a distinct and poorly immunogenic O-antigen offered no protection against colonization.

**Conclusions:**

This study suggests that O-antigen variation was introduced to the classical *bordetellae* via HGT through recombination. Additionally, genetic variation may be maintained within the O-antigen locus because it can provide escape from immunity to different O-antigen types, potentially allowing for the circulation of different *Bordetella* strains within the same host population.

## Background

The classical *Bordetella* subspecies, *B. bronchiseptica, B. parapertussis,* and *B. pertussis*, are very closely related (> 95% DNA sequence identity), but have diverged via large scale DNA loss (up to 25% of genome), or recombination [1,2]. *B. bronchiseptica* isolates retain a larger genome, the ability to grow efficiently in environmental reservoirs, such as lake water, and also infect a wide-range of mammals, including immuno-deficient humans [1,2]. Disease severities can range from asymptomatic carriage to lethal pneumonia [3], but in general *B. bronchiseptica* infections are lifelong and benign [2]. *B. parapertussis* and *B. pertussis*, the causative agents of Whooping Cough in humans, appear to have independently evolved from a *B. bronchiseptica*-like progenitor by loss of many genomic regions accompanying their adaptation to a closed life cycle, spreading from human to human without an environmental reservoir [2,4]. Many genes involved in environmental survival have been lost in the human adapted subspecies, and genes involved in infection are largely retained but differentially expressed [4]. Importantly, there are no known examples of genes acquired horizontally that contribute to the differential infections caused by these organisms [4]. The differences in infection phenotypes of the classical *Bordetella* subspecies have been related to their differential expression of a largely shared set of virulence factor genes, rather than acquisition of new genes [4]. Intriguingly, our recent comparative analysis of genomes of diverse bordetellae strains revealed that the classical bordetellae pan-genome is open, but with little uptake of new genetic material [5]. Although there are few in depth analyses on individual bordetellae loci to determine mechanisms or the selective pressures contributing to variation, previous analysis has shown some evidence for HGT in several loci shared by most strains, such as the Pertussis Toxin assembly locus [5]. In the previous analysis, one such additional locus predicted to be horizontally transferred was that encoding the O-antigen.

A component of the lipopolysaccharide (LPS), O-antigen is an important Gram-negative factor that protects against innate immunity, blocks antibody binding, and provides protection against environmental stresses, such as antibiotics [6]. There is considerable antigenic variation among O-antigens within and between bacterial species, including differences in sugar composition, chain length, and linkages due to transfer of the entire cluster of O-antigen genes or portions of the locus [7]. For example, *Escherichia coli* (*E. coli*) have over 170 antigenically distinct O-antigens, which contribute to evasion of the immune response [8]. Other bacterial species, such as *Burkholderia pseudomallei*, have less variation with two O-antigen serotypes identified [9]. However, there is still little understanding of why some organisms exchange DNA frequently resulting in increased variation, while others add DNA rarely or never, and of the evolutionary pressures that affect the frequency of HGT in each specific bacterial species.

Although the classical *Bordetella* subspecies share many known antigens that can induce cross-reactive antibodies, their LPS structures differ in ways that may be important to their overall cross-immunity. In *B. bronchiseptica,* the LPS is comprised of Lipid A, an inner core (Band B), an outer core trisaccharide (Band A) and O-antigen encoded by *lpx, waa, wlb,* and *wbm* loci, respectively [10]. The architecture of the LPS amongst the species is similar in its acylated Lipid A and branched-chain core oligosaccharide, although there are marked differences in acylation patterns of the Lipid A between all three subspecies [11,12]. In addition, several strains of *B. parapertussis* do not produce the trisaccharide, likely due to a mutation in the *wlb* locus, while *B. pertussis* does not produce an O-antigen due to the lack of the *wbm* locus [10,13]. The O-antigen locus in most *B. bronchiseptica* and *B. parapertussis* strains contains 24 genes, while the recently characterized *wbm* locus of one *B. bronchiseptica* strain (MO149) contains only 15 genes, most of which are genetically divergent from the previously characterized loci [1,4,10,14]. The first 14 genes within the O-antigen locus are thought to be responsible for the biosynthesis of the pentasaccharide linker region connecting the O-polysaccharide to the inner core, synthesis of the polymer subunit, and the capping sugar [10,11,15]. Specifically, genes within the middle of the O-antigen locus are predicted to be responsible for modifications of the terminal sugar residue [1,11]. Two sets of modifications have been noted to correlate with O1 and O2-serotypes, suggesting that antibodies against the O-antigen are directed against these terminal modifications [15-17]. Additionally, it has been shown that O1-specific immune serum does not recognize O2-specific O-antigen molecules and vice versa, suggesting that varying antigenicity could allow for evasion of existing immunity within hosts [17].

This study builds on preliminary evidence of HGT within the O-antigen loci of several newly sequenced *Bordetella* strains [5]. We observed distinct regions within the locus with lower GC content and greater sequence diversity (<50% sequence similarity), but low dN/dS ratios than the rest of the genomes. In addition, incongruent branching patterns were observed in a phylogenetic tree based on variable genes in the locus compared to a genome-wide SNP tree, suggesting that HGT may have occurred within regions of this locus. Furthermore, there appears to be extensive recombination in regions within the locus. Since HGT appears to be rare amongst bordetellae, we hypothesized that repeated HGT within this locus could be the result of strong selective pressure for escape from immunity to the parental strain’s O-antigen type. *B. bronchiseptica* strains induced antibodies that efficiently recognized and killed bacteria with the same O-antigen type *in vitro,* and *in vivo* protected against infection in mice. However, strains of one O-antigen type were largely unaffected by immunity generated against strains of other O-antigen types. Overall, this work describes evidence of multiple HGT events within regions of the O-antigen locus via recombination, and suggests escape from host immunity as a pressure that could select for these HGT events in the classical bordetellae.

## Results

### Diversity within the wbm loci correlates with different O-antigen immunogenic types

**Figure 1:**
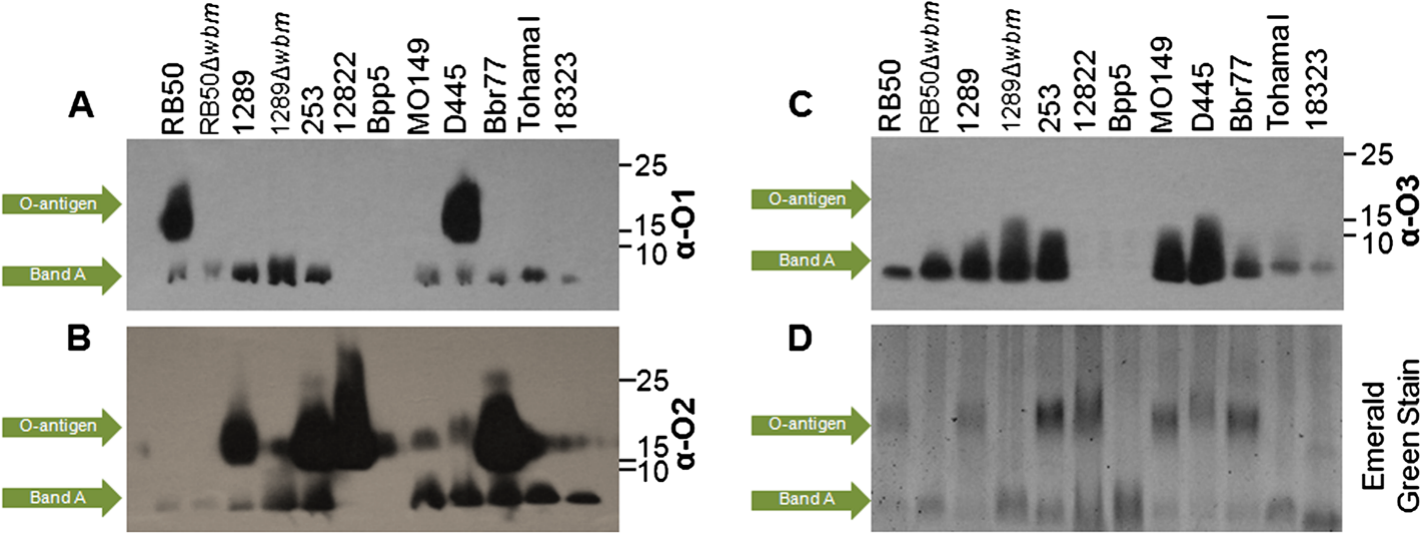
**O-antigen type of the *Bordetella *species is either O1, O2, or O3. (A-C)** C57BL/6 mice were inoculated with 10^4^CFU *B. bronchiseptica* strains RB50 (O1), 1289 (O2) or MO149 (O3), and serum was collected 28days later. LPS or LOS was purified from the indicated strains and were probed with serum against the aforementioned bordetellae. **(D)** LPS or LOS was stained with Emerald green; O-antigen and Band A are denoted.

*Bordetella* O-antigen types were previously defined as O1- or O2- serotype based on cross-reactive antigenicity [17], but recently it was shown that *B. bronchiseptica* strain MO149 produces a poorly immunogenic O-antigen designated O3 [14]. This O-antigen, unlike O1 and O2, was not recognized by antibodies generated during infection, and did not cross-react with any *Bordetella* O1- or O2- serotype specific antibodies [14-16]. To assess the diversity of O-antigen antigenicity among the classical bordetellae, we examined more strains by purifying their LPS and probing with antibodies generated against LPS containing either O1-, O2-, or O3-type O-antigens (*B. bronchiseptica* strain RB50, *B. bronchiseptica* strain 1289, or *B. bronchiseptica* strain MO149, respectively) (Figure 1A-C). Among the newly sequenced strains, *B. bronchiseptica* strain D445 was recognized by O1-specific serum, while other strains (*B. bronchiseptica* strains 253 and Bbr77) were recognized by O2-induced serum. As indicated by Emerald Green stain of the LPS, both *B. pertussis* strains Tohama I and 18323, as well as *B. parapertussis*_*ov*_ strain Bpp5, do not produce detectable O-antigens (Figure 1D).

**Figure 2:**
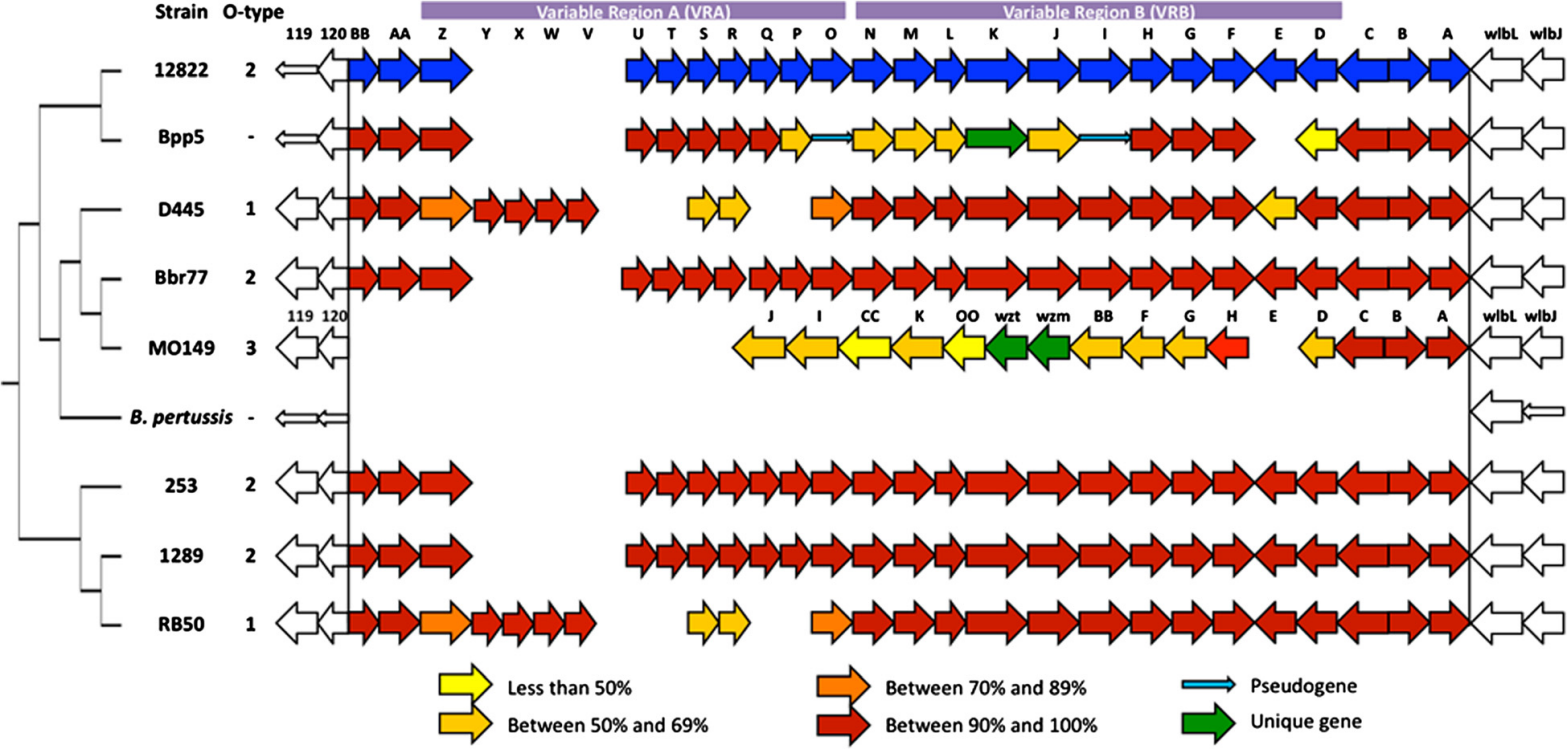
**The O-antigen loci among the classical bordetellae.** The O-antigen loci from the sequenced classical bordetellae strains with several flanking genes were displayed in this figure. Sequence similarity based on 12822 (blue) was shown with different color schemes based on their % identity.

**Figure 3:**
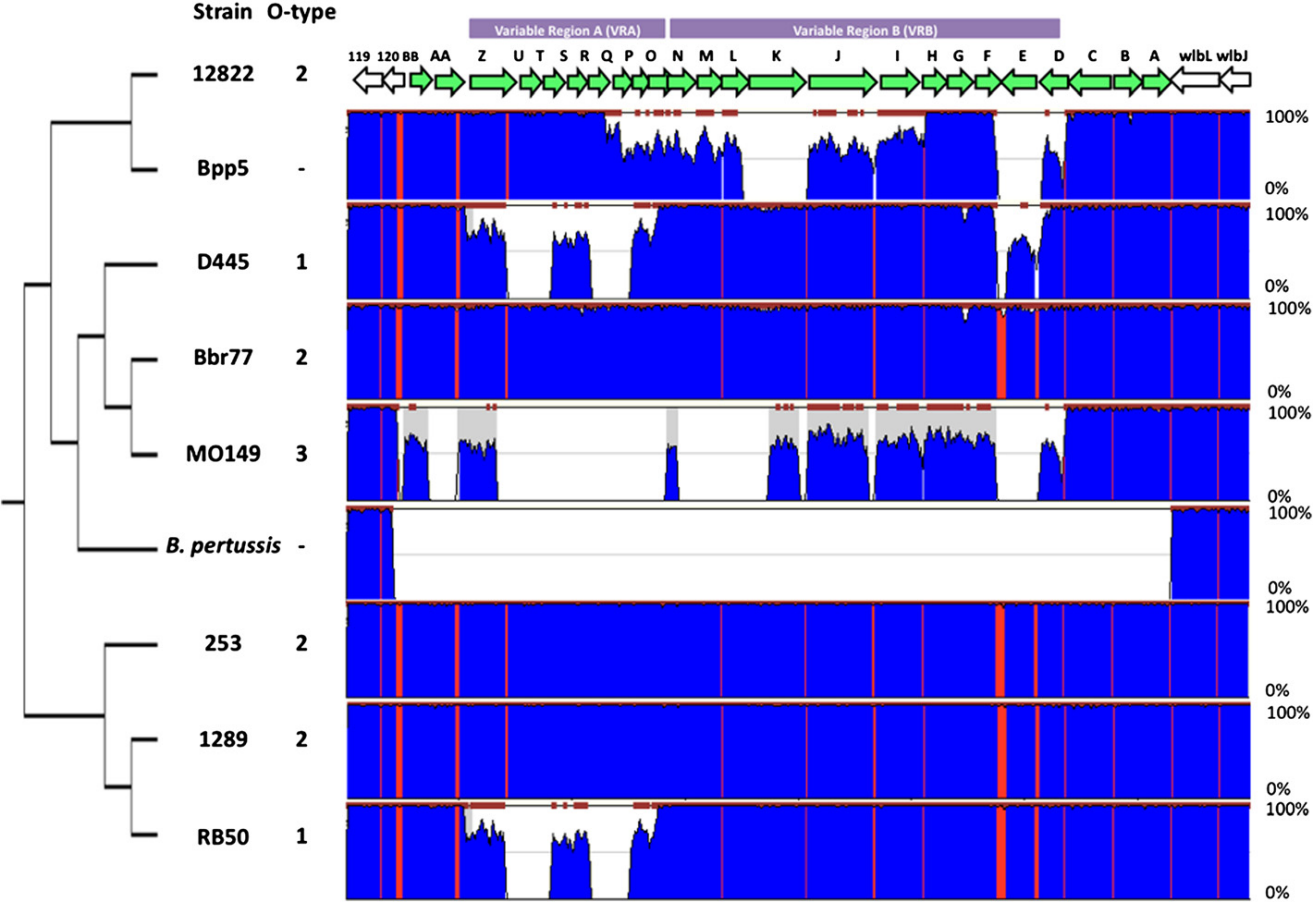
**Sequence similarity across the classical bordetellae O-antigen loci compared to *B. parapertussis *12822.** Percent similarity of the O-antigen locus and flanking genes based on 12822 was plotted between 0% and 100% using zPicture [42]. Intergenic regions and coding regions were highlighted with red and blue, respectively. The genome-wide SNP tree was superimposed on the left.

Previous analysis of the first three *Bordetella* genomes revealed differences between *B. bronchiseptica* strain RB50 and *B. parapertussis*_*hu*_ strain 12822 within a region of the O-antigen locus shown to be required for O-antigen expression, *wbmZ*-*wbmO*[4,10,15-17]. Additionally, previous Comparative Genomic Hybridization (CGH) analysis revealed variability across the locus in many lineages [1]. To define the changes that have arisen within the O-antigen locus since the recent divergence of these organisms, we compared the sequence of the entire *wbm* locus and flanking genes in 10 genomes and plotted the percent nucleotide identity of these genes in each strain, including the seven newly sequenced genomes, to that of *B. parapertussis*_*hu*_ strain 12822 (Figures 2 and 3). Genes flanking this locus appear to be as highly conserved as the rest of the genomes of these closely related strains. Both *B. pertussis* isolates (18323 and Tohama I) lack the *wbm* locus and make no O-antigen, suggesting the locus was lost during their relatively recent divergence from the other classical *Bordetella* subspecies. The remaining eight strains all have highly conserved genes at either end of the *wbm* locus as well as multiple other genes within the locus that are conserved and apparently intact, and likely functional. This is consistent with the >95% identity in the core genomes of even the most distantly related of these strains (unpublished data). However, only three strains, Bbr77, 253 and 1289, contain an intact set of orthologs of all the *wbm* genes of the reference *B. parapertussis* strain 12822 and the LPSs of all four strains with this set of genes are recognized by anti-O2 O-antigen type antibodies (Figure 1B). Interestingly, these four strains are distributed widely across a genome-wide SNP-based phylogenetic tree (Figure 2) [5]. The two O1-type strains, RB50 and D445, are similar to the four O2 strains in their conservation of the 14 genes from *wbmA* through *wbmN*, but contain a very different set of genes within what we have designated Variable Region A (VRA) that correlates with their antigenically distinct O1 type (*wbmO-wbmZ)*. A comparison of all ten strains using RB50 as the reference reveals that D445 is >95% homologous to RB50 across the entire locus except for a small region comprising *wbmE* (see Additional file 1). The two remaining strains, Bpp5 and MO149, both differ from the other six strains in a region we have designated Variable Region B (VRB) (Figure 2). Strain MO149, the sole example of the poorly antigenic O3 type (Figure 1C and D) [14], lacks VRA genes and has a set of genes in VRB that appear intact, but are either novel or highly divergent orthologs with as little as 50% similarity to reference strain genes (Figure 2). This set of genes is apparently sufficient to make a poorly antigenic O-antigen (Figure 1C and D) [14]. Strain Bpp5, which does not appear to make O-antigen, has a nearly complete VRA, but a highly divergent VRB, with several genes of low similarity to reference genes, one unique gene and two apparent pseudogenes. The two variable regions (VRA and VRB) are flanked in all *B. bronchiseptica* and *B. parapertussis* strains by genes *wbmA, wbmB* and *wbmC*, which are conserved and share between 90 and 100% sequence similarity (Figure 2). In addition, all of these strains except MO149 also share highly conserved genes *wbmAA* and *wbmBB* on the other flank. Interestingly, some distantly related strains retain highly homologous genes to the reference (i.e. *B*_*.*_*bronchiseptica* strain 1289), while other strains that are much more closely related differ substantially in discrete islands of genes. For example, *B. parapertussis* strains isolated from human (12822) and sheep (Bpp5) differ in two distinct regions, *wbmI-Q* and *wbmD-F* (Figure 3). Also, some strains are closely related based on genome-wide SNPs (i.e. 1289 and RB50) but have different sets of genes that correspond with their different serotypes (O1 and O2, respectively), whereas some distantly related strains (i.e. RB50 and D445) share the same serotype specific genes (O1). Together, these data reveal that eight out of ten closely related strains retain genes of high sequence similarity within this locus, but that four of these strains differ substantially in one or both of two variable regions. Overall, these differences appear to correlate with antigenically different O-antigen types, but do not correspond to the genome-wide SNP-based phylogenetic tree relating these strains.

### Horizontal gene transfer of O-antigen loci in the classical bordetellae

**Figure 4:**
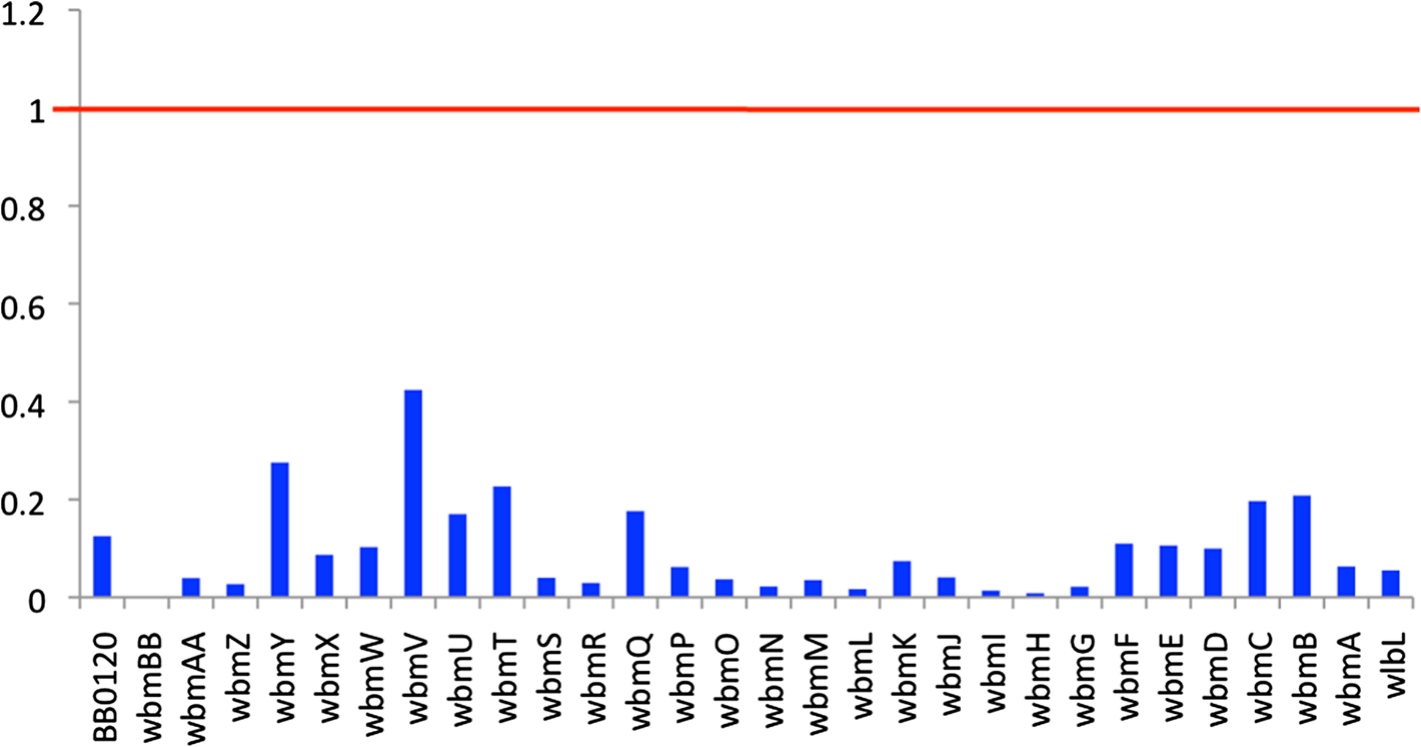
**dN/dS ratios of the genes within the O-antigen loci.** dN/dS ratios for the genes in the O-antigen loci of all the strains were calculated using PAML package with the Nei-Gojobori method.

One potential explanation for high SNP density within a region is that spontaneous mutations accumulate due to positive (diversifying) selection. To examine positive or negative selective pressures on the evolution of the O-antigen locus, we calculated the dN/dS ratio for each gene across the entire locus for all strains (Figure 4). For all genes within all strains, the *wbm* loci dN/dS ratios were below 1, a signature of negative (purifying) selection, indicating that positive selection is not the driving evolutionary force (Figure 4 and see Additional file 2). We also found no positively selected sites (Posterior probability>0.95) in any of genes, except *wbmF* and *wbmC* (see Additional file 3). However, when we account for recombination using PARRIS in HyPhy Package [18], there is no evidence of positive selection in either *wbmF* or *wbmC* (see Additional file 4), suggesting the possibility of other mechanisms, such as HGT, as the source of the diversity of the O-antigen locus.

**Figure 5:**
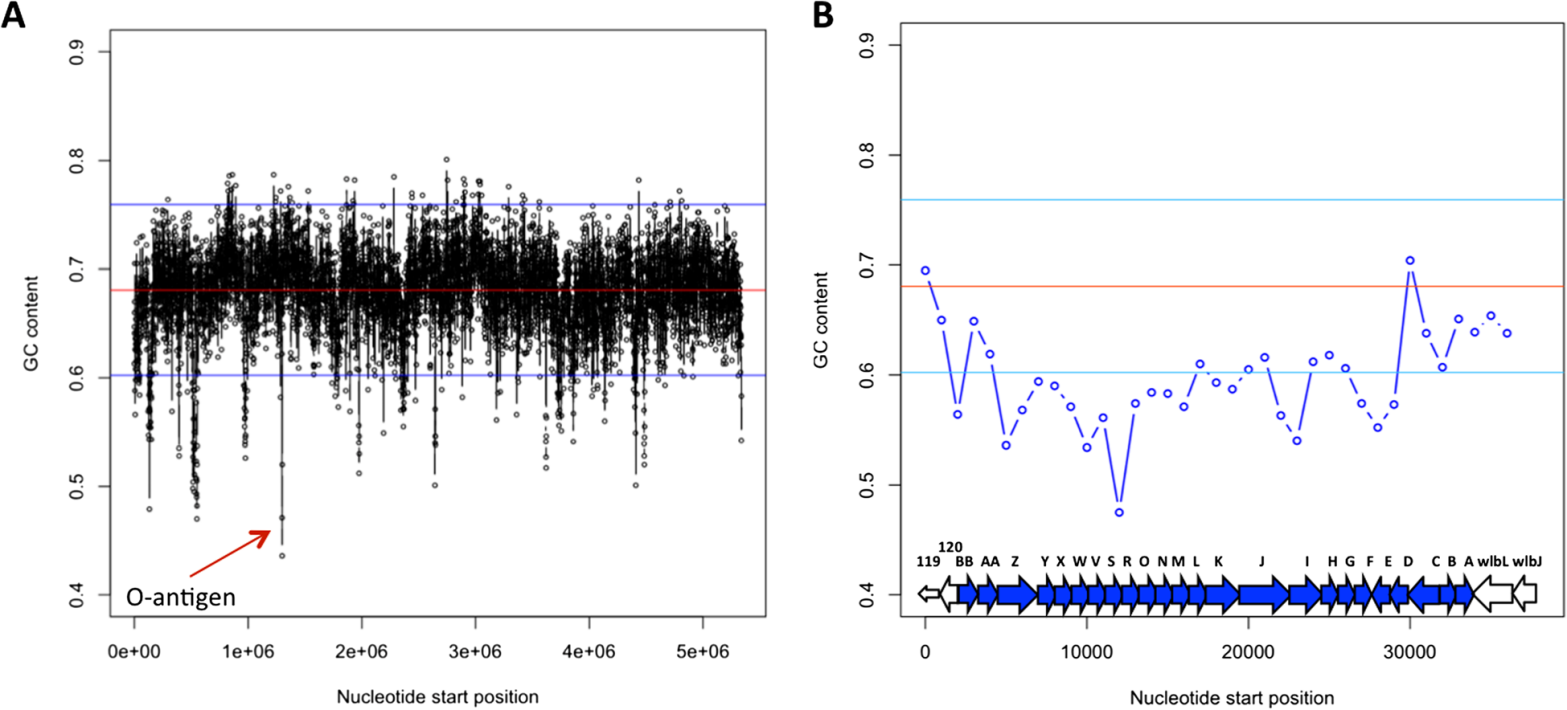
**GC content of *B. bronchiseptica *strain RB50.** The genome-wide **(A)** or O-antigen locus **(B)** GC content of *B. bronchiseptica* strain RB50 is plotted in 1,000 base pair increments. The overall average GC content of the entire genome is indicated by the red line, and the blue lines represent the standard deviation across the genome.

One signature of HGT is that the acquired region displays a difference in genome-wide characteristics, such as GC content. As previously noted in other bacterial species where O-antigen loci have been transferred GC content is consistently lower than the rest of the genome [7]. An analysis of the GC content of the entire bordetellae genomes identified the O-antigen locus as the most different from the genome average GC content of ~67% (Figure 5 and see Additional file 5), with the GC content of many *wbm* genes differing by more than two Standard Deviations (SD) from the genome average. Similar results were observed using a 1,000 base pair sliding window (Figure 5B and see Additional file 5). Strains of the same O-antigen type had patterns of low GC content that were similar to each other, but differed from those of other O-antigen types (see Additional file 5). Intriguingly, the lowest GC content corresponded to the regions of highest diversity within VRA and VRB (see Additional file 5), suggesting the evolutionary history that gave rise to this diversity was not consistently under the pressures that maintain very high GC content elsewhere in the genomes of the bordetellae. These data also suggest the possibility of HGT of the locus.

**Figure 6:**
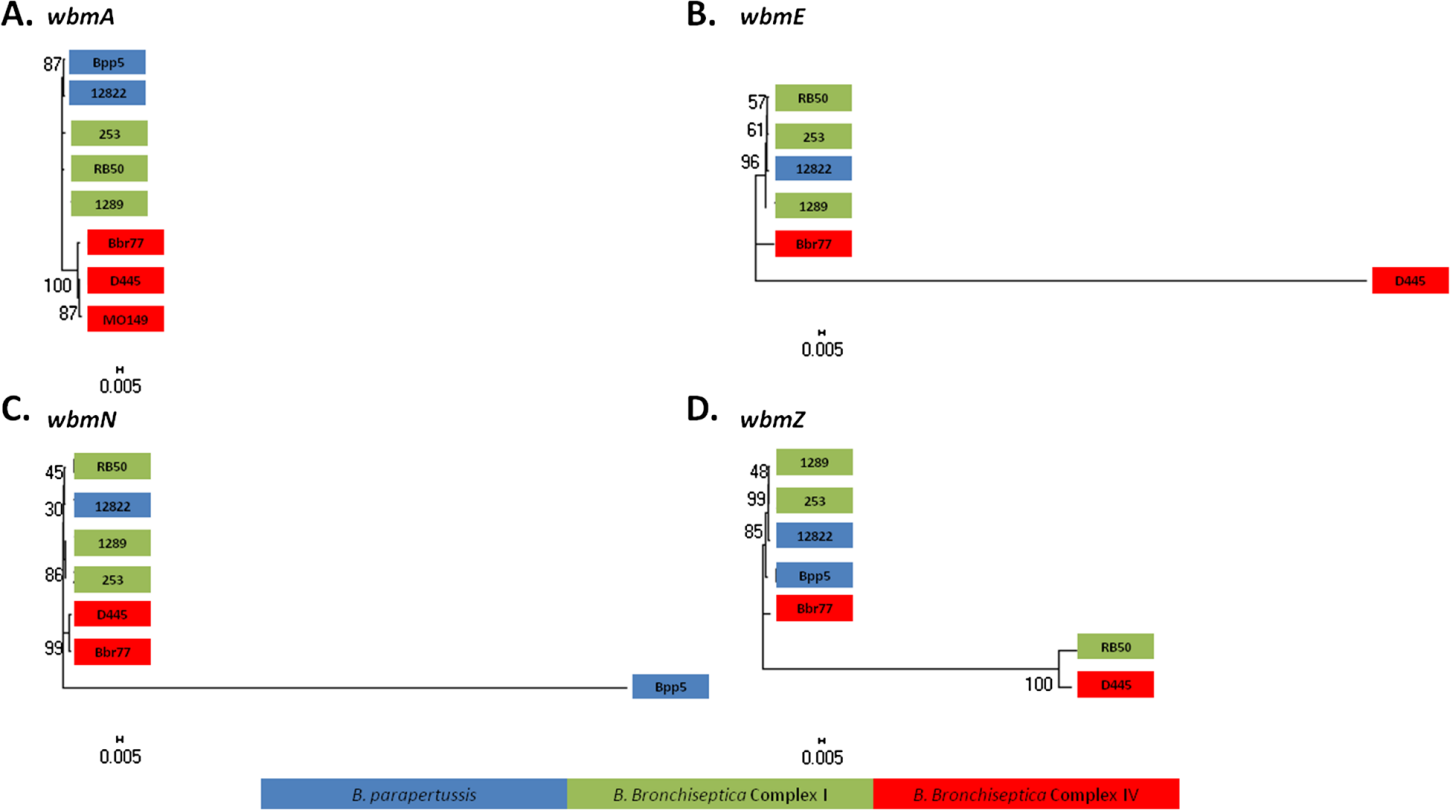
**Phylogenetic trees for the classical bordetellae O-antigen loci.** Phylogenetic trees were constructed by maximum likelihood method with 1,000 bootstrap replicates with individual gene sequences in the locus. Four representative (**A**: *wbmA,***B**: *wbmE,***C**: *wbmN,* and **D**: *wbmZ*) of similar phylogenetic trees were presented in this figure.

To further assess the likelihood of HGT within VRA and VRB of the O-antigen locus, we constructed maximum-likelihood trees based on individual O-antigen genes and compared them with the genome-wide SNP tree (Figure 6). Trees based on conserved genes, such as *wbmA,* were similar to the genome-wide SNP tree and the previous multi-locus sequencing typing (MLST) tree [1], with *B. bronchiseptica* complex I and IV strains clustering separately, for example. Genes within VRA, including *wbmZ*, produced trees with different branch patterns that correlated with O-serotype, further supporting the conclusion that these genes are likely to have been laterally transferred (Figure 6 and see Additional file 6). Additionally, other genes, such as *wbmL* and *wbmE*, within the VRB produced trees with isolated branches containing either one or two strains (*B. parapertussis*_*ov*_ strain Bpp5, *B. bronchiseptica* strains MO149 or D445), while other strains appeared to cluster together with a branching pattern similar to the genome-wide SNP tree (Figures 2, 3, and 6 and see Additional file 6). These data are consistent with the conclusion that genes within the O-antigen locus were horizontally transferred.

### Horizontal gene transfer via recombination

**Figure 7:**
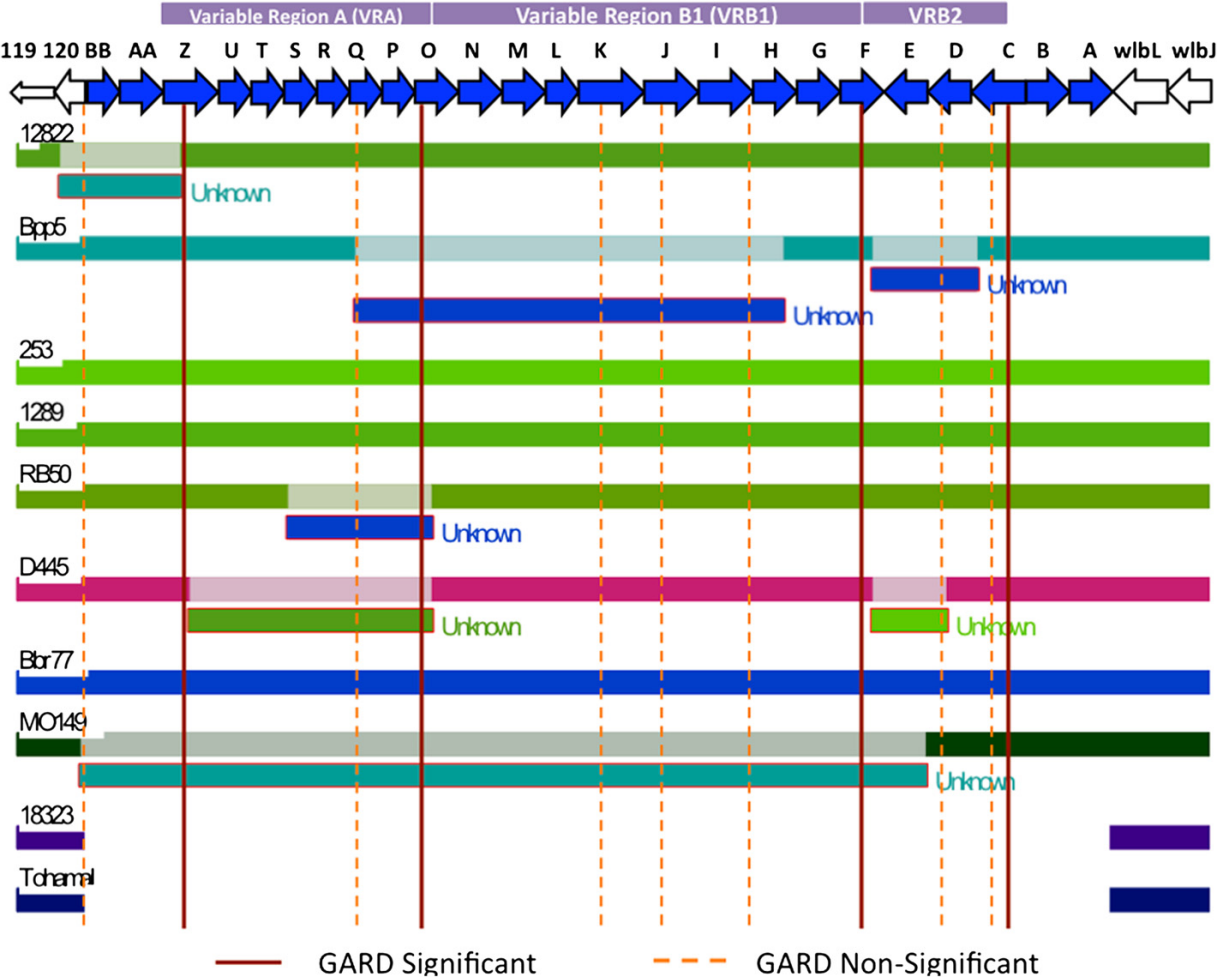
**Predicted recombination breakpoints for the classical bordetellae O-antigen loci.** The recombination breakpoints are predicted via Recombination Detection Program (RDP) and Genetic Algorithms for Recombination Detection (GARD) in all the classical bordetellae strains. Major breakpoints are indicated by a solid line while minor break points are indicated by a dashed line.

Since we did not observe any mobile elements associated with HGT, we used a multi-software analysis approach to predict recombination breakpoints within the locus. We used both substitutions-distribution methods, such as GENECONV, SiScan, MaxChi, and Chimaera, which test for significant substitutions clustering within sequences, and phylogenetic methods, including Recombination Detection Program (RDP), BootScan, and Genetic Algorithms for Recombination Detection (GARD), which search for significant differences in tree topologies in order to predict recombination breakpoints [19]. Both methods predicted several recombination breakpoints in the O-antigen locus (Figure 7). Although there were some differences in the exact positions identified by each method, we observed similar trends overall. From the GARD method, there were 4 major breakpoints, identifying 5 different segments (*wbmBB*-*wbmZ*, *wbmZ*-*wbmO,wbmO*-*wbmF*, *wbmF*-*wbmC*, and *wbmC*-*wbmA*) in the O-antigen locus (Figure 7, solid lines). The *wbmZ-wbmO* gene cluster (VRA) correlates with serotypes O1 and O2. Within VRB, two segments were predicted in this recombination analysis, VRB1 (*wbmO-wbmF*) and VRB2 (*wbmF-wbmC*). Additionally, there were some minor breakpoints in BB0120, *wbmQ*, *wbmK, wbmJ, wbmI/H, wbmD,* and *wbmC* (Figure 7, dashed lines), most of which are in VRA or VRB. From seven different methods in the RDP3 package, seven segments were predicted, detected by more than four methods in RDP, in the different strains. In *B. bronchiseptica* strain MO149, recombination breakpoints were predicted in BB0120, and between *wbmE* and *wbmD*. Two segments were predicted in *B. bronchiseptica* strain D445, including *wbmZ*-*wbmO* and *wbmF*-*wbmD,* while one segment between *wbmS/T* and *wbmO* was predicted in *B. bronchiseptica* strain RB50. Additionally, two fragments were predicted in *B. parapertussis*_*ov*_ strain Bpp5, including regions between *wbmQ* and *wbmH* as well as between *wbmF* and *wbmC*. Lastly, one segment between BB0119 and *wbmZ* was predicted in *B. parapertussis*_*hu*_ 12822. These segments appeared to be transferred from unknown sources, although most of the segments have high similarity to regions within other bordetellae strains (Figure 7). Interestingly, our previous phylogenetic analysis was supported by this recombination analysis. For example, we observed different tree topology trends in conserved regions at the beginning and end of the locus as well as in VRA and VRB (Figure 6). These topology trends correlate with the five observed consensus segments (*wbmBB*-*wbmZ*, **VRA:***wbmZ*-*wbmO,***VRB1:***wbmO*-*wbmF*, **VRB2:***wbmF*-*wbmC*, and *wbmC*-*wbmA*) in our recombination analysis (Figure 7). Together, these data suggest that parts of the O-antigen locus may have been horizontally transferred via homologous recombination.

### Selective advantage of the divergent O-antigens in the bordetellae

**Figure 8:**
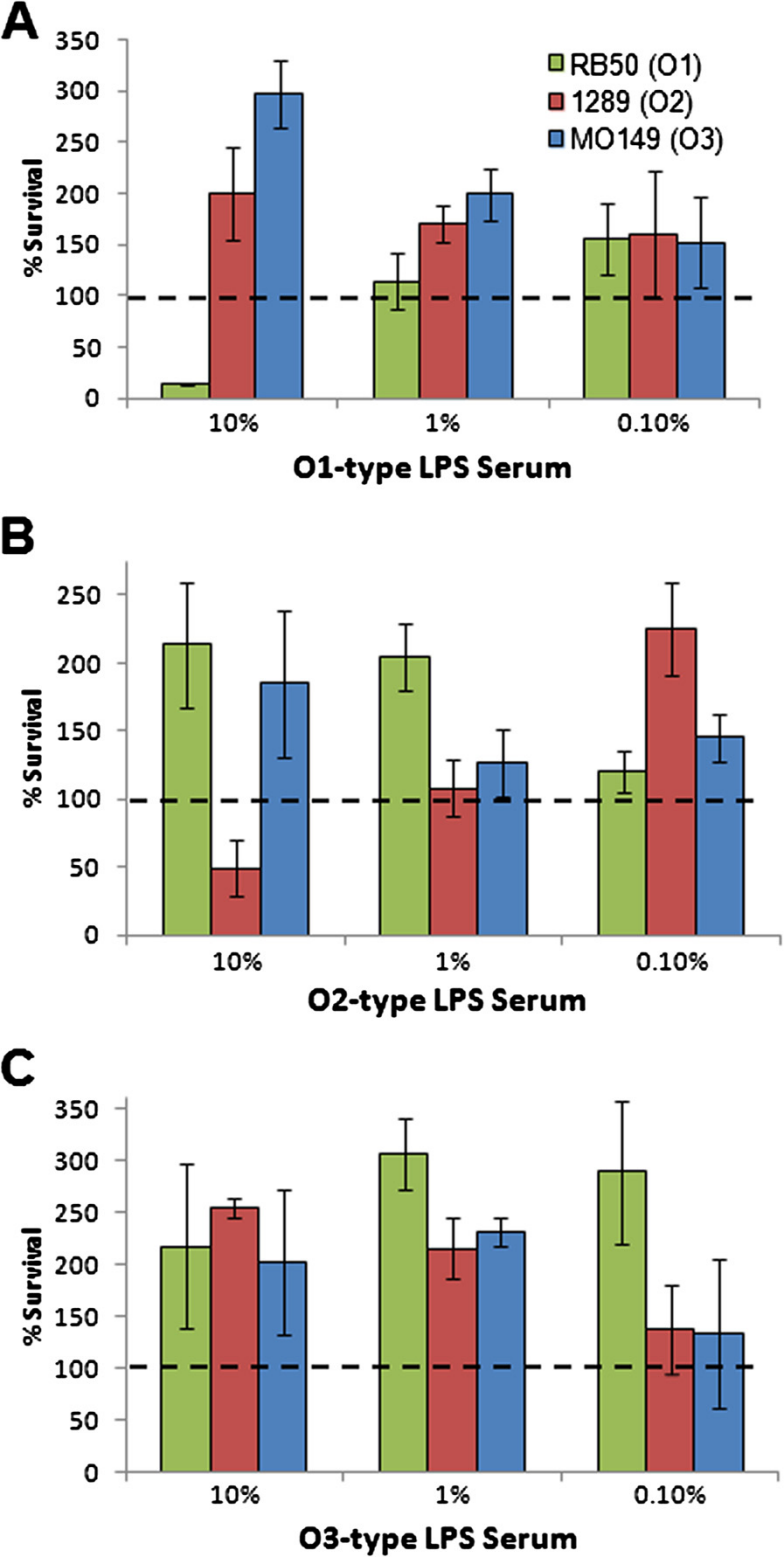
**Immune mediated serum killing of O1, O2 or O3 type LPS.***B.bronchiseptica* strains RB50 (green), 1289 (red) or MO140 (blue) were incubated with convalescent immune serum from C57/BL6 mice inoculated with 10^4^ of O1-type LPS **(A)**, O2-type LPS **(B)** or O3-type LPS **(C)** at the indicated serum percentages in the presence of naïve serum. Dashed line indicates 100% survival of bacteria.

Since HGT appears to be rare amongst *Bordetella* subspecies, evidence of multiple HGT events in the O-antigen locus could reflect an increased frequency of acquisition of the genes within this locus or selective pressure on these genes. To examine the potential selective advantage of acquiring a new O-antigen serotype, we incubated *B. bronchiseptica* strains RB50 (O1-type), 1289 (O2-type) or MO149 (O3-type) with serum containing antibodies generated against each LPS type. 10% O1 LPS-specific serum killed >85% of O1 strain (RB50), but did not kill either O2 (1289) or O3 (MO149) strains (Figure 8A). This antibody-mediated killing was dose-dependent, as 1% and 0.1% O1-specific serum did not kill strain RB50 (Figure 8A). In a similar dose-dependent manner, O2 LPS-specific serum killed the O2 strain but did not kill either O1 or O3 strains (Figure 8B). Surprisingly, O3 LPS-specific serum antibodies did not kill any of these strains (Figure 8C). Thus, antibodies generated by non-cross reactive O-antigen types cannot recognize and kill other strains with different O-types, and the O3 serotype does not appear to induce an effective O-antigen antibody response.

**Figure 9:**
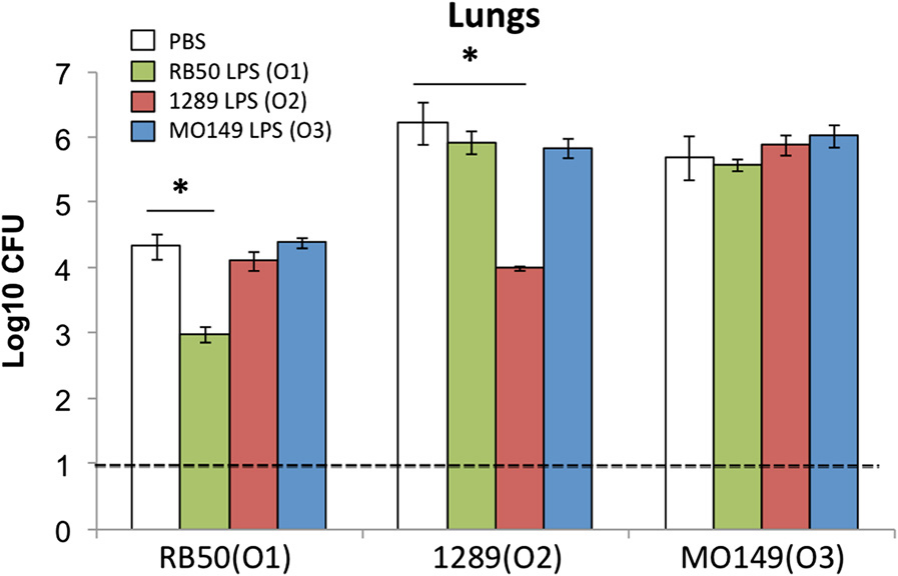
**O1, O2, or O3 LPS vaccination affects colonization of *B. bronchiseptica *strains.** C57/BL6 mice were vaccinated with purified LPS (100 ng/per mouse) from RB50 (green), 1289 (red), MO149 (blue), or were sham vaccinated with PBS (white) on days 0 and 14. On day 28, mice were inoculated with 10^4^ CFU of RB50, 1289 or MO149, and lung colonization was determined 3 days post-inoculation. The error bars represent standard deviation of 4 mice per group. * indicates a p value <0.05.

In order to assess if an antigenically different O-antigen serotype could confer a selective advantage by allowing evasion of immunity to other O-antigen serotypes, mice were vaccinated with different O-type LPSs and challenged with O1-, O2-, or O3-type bacteria. Previous research indicated that O1 and O2-type O-antigens do not induce cross-protective antibodies that mediate efficient clearance of bacteria with the opposite O-antigen type [17]. Similar to previous results, nasal cavity and tracheal colonization was not affected by any LPS vaccination (see Additional file 7). However, O1-type LPS vaccination reduced RB50 (O1-type) colonization in the lungs by ~90%, but colonization by 1289 (O2-type) or MO149 (O3-type) was unaffected (Figure 9). Similarly, vaccination with O2-type LPS reduced lung colonization by an O2 strain by 99%, but did not affect colonization by O1 or O3 strains. Strikingly, O3-type LPS vaccination did not reduce colonization by any *B. bronchiseptica* strain, consistent with this O-type being poorly immunogenic. Overall, these results indicate different O-antigens induce little cross-protective immunity, suggesting that acquisition of a new O-antigen type via HGT could confer a selective advantage.

## Discussion

The classic examples of HGT involve the acquisition of clusters of new genes to a species, such as plasmids or pathogenicity islands, via mechanisms involving insertion sequences or other mobile elements [20,21]. While acquiring new genes by HGT is often associated with changes in bacterial pathogenesis, within the classical bordetellae subspecies these differing characteristics have not been attributable to the acquisition of any new genes conferring differential host specificity or increased pathogenesis [4]. Intriguingly, our recent analysis of the genomes of eleven diverse strains of classical *Bordetella* revealed substantial genome loss in some lineages, but no known acquisition of new genes that could be associated with differing phenotypes [5]. This analysis based on the aforementioned genomes also predicted that the pan-genome of the classical bordetellae was open with limited uptake of new genetic material. The multiple recombination breakpoints within the O-antigen locus appear to represent HGT events within the *Bordetella* evolutionary history and subsequent exchange between bordetellae subspecies (Figure 7). These organisms therefore represent a model system to understand how pathogens evolve over time, as well as present the opportunity to examine pressures that drive selection for HGT events.

HGT between closely related bacterial species is more frequent, albeit harder to detect, than between distantly related bacteria [20,22]. This may be because closely related species are in greater contact in that they share overlapping ecological niches or because they have similar genomic characteristics, making transferred DNA less likely to be rejected [22]. Based on low GC content, high SNP densities with low dN/dS ratios, and dissimilar phylogenetic trees across the locus, we identified subsets of genes within the O-antigen locus of *B. bronchiseptica* strains, including genes encoding O1 and O2-types, that appear to have been acquired via HGT. Although we have not yet identified any associated mobile elements, such as a tRNA [23] or insertion sequence elements [24], we have predicted several recombination breakpoints within the locus. In *Salmonella enterica*, *Escherichia coli,* and *Klebsiella* species, genes toward the center of the O-antigen cluster are less conserved compared to the genes at the ends, and this has been suggested to facilitate the exchange of interchangeable modules within the locus to form new O-antigens [6]. The absence of mobile elements near the breakpoints suggests multiple recombination-mediated HGT events involving O-antigen genes contribute to the different O-antigen types of the classical bordetellae subspecies. Previous analysis of classical bordetellae genomes indicates that these bacterial subspecies, particularly *B. pertussis*, have evolved through genome loss, which is associated with adaptation to a closed lifestyle within its human host [4]. Notably, *B. bronchiseptica* complex IV strain MO149, isolated from a human, produces the poorly immunogenic O-antigen that shows evidence of recombination based HGT, as well as has lost several genes within the locus. Therefore, it is also possible that variation within the O3-type locus may be indicative of gene acquisition or the genome undergoing reduction as *B. bronchiseptica* complex IV strains potentially adapt to a new environment.

The functional interdependence of the O-antigen synthesis/assembly pathways appears to limit recombination within the classical bordetellae locus. For example, the segment between *wbmA* and *wbmC*, which was predicted to contribute to the linker region synthesis [14], is confirmed to be conserved as a unit (Figure 2). Since *wbmF, wbmG,* and *wbmH* together constitute the pathway for converting UDP-ManNAc3NAcA to the UDP-GalNAc3NAcA for the O PS backbone [25], they also appear to be conserved as one segment. Similarly, *wbmL, wbmM,* and *wbmN*, which are within the same segment (VRB), are known to encode proteins that comprise ATP-binding cassette (ABC) transporter systems to export the O-antigen [10]. The interdependence of genes in these pathways is a critical consideration, since strains recombining in these regions might have a fitness disadvantage and thus be outcompeted. This interdependence also means that an acquired set of genes must be complete and sufficient for a new phenotype, such as an altered O-antigen serotype, in order for there to be selective advantage to its acquisition. Since HGT appears to be quite rare amongst these organisms, it is therefore of great interest that there are potentially at least five different HGT events within this locus in these ten strains. Together, these data suggest that there is strong selection for the newly acquired genes within this locus, and therefore that each set of acquired genes is sufficient to confer a novel and important phenotype.

Pan-genome analysis indicates that genes associated with diverse phenotypes, antibiotic resistance, and that confer selective advantages are often found within the accessory genome rather than the core genome [26,27]. O-antigen, which is included in the classical bordetellae accessory genome, may be variable within the subspecies due to its selective advantage of functional interdependence, immunity evasion, or host adaptation. In *S. enterica* and *E. coli,* multiple O-antigen serotypes have been identified [28-30] and are hypothesized to contribute to evasion of cross-immunity [31,32], thereby allowing the circulation of multiple closely related strains with different O-antigens within the same population. Loss of the O-antigen locus by *B. pertussis* has been suggested to be a result of competition between it and a *B. bronchiseptica*-like ancestor, which may have circulated within the human population prior to the emergence of *B. pertussis*[33]*.* Additionally, previous research investigating cross-protective immunity between O1 and O2-type O-antigens has indicated that lack of protection may allow for the circulation of antigenically distinct *B. bronchiseptica* isolates within populations [17]. The loss of antigenicity in some *B. bronchiseptica* complex IV strains, which have been suggested to be more frequently associated with human infections than complex I strains [1], may in part be due to immune-mediated competition with *B. parapertussis* in the human population. Additionally, the discovery of the third poorly immunogenic O-antigen type further highlights the significance of the immune response as a likely selective pressure that could be driving HGT amongst classical *Bordetella*. It is intriguing that the poorly immunogenic O-antigen appears to be specific to *B. bronchiseptica* complex IV isolates and is not prevalent throughout all *B. bronchiseptica* and *B. parapertussis* isolates. This may be indicative of additional functions of the O-antigen to evade host immunity, such as complement deposition [34], or perhaps evasion of phage, as has been shown in *Vibrio cholerae*[35]. Poorly immunogenic O-antigens may render *B. bronchiseptica* isolates more susceptible, whereas O1-and O2-types may be more robust at protecting these bacteria against other environmental factors.

## Conclusions

Our study suggests that HGT of portions of the O-antigen locus in *Bordetella* subspecies, mediated by homologous recombination, is a mechanism to generate divergent O-antigens. The lack of cross-immunity between different O-antigen serotypes may provide an advantage to the acquisition of a new O-antigen type and could explain the high frequency of HGT within this locus, relative to elsewhere in the genomes of *Bordetella* subspecies. The evidence that O-antigen differences may allow evasion of immunity also leads to the prediction that greater variation will be observed within this locus, relative to others, as more *Bordetella* genomes are sequenced.

## Methods

### Ethics statement

All experiments in this study were carried out in accordance with the National Institute of Health’s recommendations set forth in the Guide for the Care and Use of Laboratory Animals. The experimental protocols were approved by the Institutional Animal Care and Use Committee at The Pennsylvania State University at University Park, PA (#31297 *Bordetella*-Host Interactions). All animals were anesthetized using isoflourane or euthanized using carbon dioxide inhalation to minimize animal suffering.

### Bacterial strains and growth

All strains used in this study have been previously described [1,4,5,14]. Bacteria were maintained on Bordet-Gengou agar (Difco, Franklin Lakes, NJ) containing 10% sheep blood (Hema Resources, Aurora OR) and 20 μg/mL streptomycin (Sigma Aldrich, St. Louis, MO). Liquid cultures were grown at 37°C overnight in a shaker to mid-log phase in Stainer-Scholte (SS) broth and heptakis [36,37].

### Lipopolysaccharide purification

LPS was purified by a modified Westphal method [38]. Briefly, 500 mL cultures were seeded with mid-log phase (0.5 OD_600nm_) bordetellae and grown in a shaking incubator at 37°C. Cultures were grown to an OD_600nm_ of 1.0. Bacterial cells were then pelleted at 500 × g and resuspended in 10 mLs of endotoxin free water. An equal volume of 90% w/v phenol was added and the samples were heated to 65°C for 1 hour with stirring. Samples were then chilled followed by centrifugation at 1,000 × g. The aqueous phase was dialyzed using 1000 molecular weight cut off dialysis membrane against ddH_2_O for 48 hours. After lyophilization, the resulting material was resuspended in Tris buffer (pH7.5) and treated with 25 μg/mL of RNase (Ambion, Austin, TX) and 100 μg/mL of DNase (Mo Bio, Carlsbad, CA). 100 μg/mL of Proteinase K (Ambion, Austin, TX) was then added. Following phenol extraction, the aqueous phase was dialyzed for 12 hours against ddH_2_O and lyophilized. Resulting LPS was suspended in endotoxin free water.

### Western blots

Purified LPS from the indicated bacterial strains were separated via sodium dodecyl sulfate-15% polyacrylamide gel electrophoresis (SDS-PAGE) and transferred electrophoretically to polyvinylidene difluoride membranes (Millipore, Bedford, MA) as previously described [14,17,39]. Membranes were probed with day 28 convalescent serum, at a 1:1000 dilution, from mice inoculated with 10^4^CFU *B. bronchiseptica* strains RB50, 1289 or MO149. Membranes were then probed with goat anti-mouse (immunoglobulin H+L) horseradish peroxidase-conjugated (1:10,000) antibody (Southern Biotech, Birmingham, AL). All membranes were visualized with ECL Western blotting detection reagents (Amersham Biosciences, Piscataway, NJ).

### Animal experiments

C57BL/6 mice were obtained from Jackson Laboratories (Bar Harbor, ME). Mice were bred in our *Bordetella*- and pathogen-free breeding rooms at The Pennsylvania State University, and all animal experiments were performed with approval and in accordance to institutional guidelines. 4 to 6 week old mice were vaccinated intraperitoneally with 200 μl of LPS (100 ng) on days 28 and 14 prior to challenge as previously defined [40]. Mice were lightly sedated with 5% isoflurane (IsoFlo, Abbott Laboratories) in oxygen, and 10^4^CFU *B. bronchiseptica* strains RB50, 1289 or MO149 were pipetted in 50 ul of phosphate-buffered saline (PBS) (Omnipur, Gibbstown, NJ) onto the external nares. This method reliably distributes the bacteria throughout the respiratory tract [39,41]. To quantify bacterial numbers, mice were sacrificed at the indicated time points and the lungs, trachea and nasal cavities were excised. Organs were then homogenized in PBS, the appropriate dilution plated on BG agar with antibiotics, and CFU determined by counting colonies. For collection of convalescent or vaccination-induced serum from mice, inoculated animals were sacrificed 28 days post-inoculation or vaccination with 100 ng of purified LPS and bled orbitally as previously described [41]. To obtain serum, blood was incubated at room temperature for 30 minutes and then spun for 5 minutes at 250 × g. Serum was collected and stored at −80°C. For all appropriate data the average +/− the standard deviation (error bars) are presented. Results were analyzed using the Student’s *t* test with a *P* value of <0.05 considered significant.

### Sequence analysis and GC content

Sequence percent similarity of the O-antigen locus of all eight previously published strains [5] and flanking regions based on *B. parapertussis* strain 12822 was plotted between 0% and 100% using zPicture [42]. GC content was calculated using the sliding window method (window size 1,000 base pairs) across the genome of *B. bronchiseptica* strain RB50 or the O-antigen locus of all eight strains using R [43]. Average and standard deviation for the genome-wide GC content were also calculated by R.

### Phylogenetic analysis and dN/dS ratios

Multiple alignments of individual genes in the O-antigen locus were generated by the MEGA5 software, and maximum likelihood trees were constructed with a Tamura-Nei model and 1,000 bootstrap replicates [44]. dN and dS values were computed using PAML package [45] with the Nei-Gojobori method [46]. In Hyphy, PARRIS was used to detect site-specific selection in *wbmF* and *wbmC,* while taking recombination and synonymous rate variation into account [18].

### Recombination analysis

The O-antigen locus nucleotide sequences were aligned based on *B. parapertussis*_*hu*_ strain 12822, using Ssaha v2.2.1 [47]. Then, both Recombination Detection Program (RDP3) [48] and Genetic Algorithm for Recombination Detection (GARD) [49] were used to detect recombination breakpoints in the O-antigen locus. In RDP3, default setting (with RDP, GENECONV, MaxChi, BootScan, and SiScan) was used, except adding two more detection methods, including Chimaera and 3Seq, and listing all recombination events. Once the analysis was complete, *B. parapertussis*_*ov*_ strain Bpp5 was corrected to be the recombinant strain based on parsimony and all the other recombination events were accepted, and then rescanned. All the predicted recombination events were detected by at least six methods in RDP3, except the last small fragment in *B. parapertussis*_*ov*_ strain Bpp5, which was detected by four methods. The same alignment was analyzed by GARD program available at the datamonkey server using HKY85 substitution model.

### Complement-killing assay

As previously described [50], bacteria were grown to mid-log phase, and 10^3^CFU were incubated in serum containing antibodies generated against either O1-type LPS, O2-type LPS or O3-type LPS at the indicated concentrations, with naïve serum, or PBS for 1hour at 37°C. Bacteria were serially diluted and plated on BG containing 25 μg/mL of streptomycin. CFU were counted and compared to the initial inoculums in order to determine percent survival of bacteria. For all appropriate data the average +/− the standard error (error bars) are presented. Results were analyzed using the Student’s *t* test with a *P* value of <0.05 considered significant.

### Availability of supporting data

The data sets supporting the results of this article are included within the article (and its Additional files).

## Competing interests

The authors declared that they have no competing interests.

## Authors’ contributions

SEH, JP, and ETH conceived and designed experiments. SEH, JP, LLG, HAF, and AP performed the experiments. SEH, JP, and ETH analyzed the data and wrote the paper. All authors read and approved the final manuscript.

## Supplementary Material

Additional file 1 **Sequence similarity across the classical bordetellae O-antigen loci compared to *B. bronchiseptica* RB50.** Percent similarity of the O-antigen locus and flanking genes based on RB50 was plotted between 0% and 100% using zPicture [42]. Intergenic regions and coding regions were highlighted with red and blue, respectively. The genome-wide SNP tree was superimposed on the left. Click here for file

Additional file 2 **Pair-wise dN/dS ratios of individual genes within the O-antigen loci.** Pair-wise dN/dS ratios for the genes in the O-antigen loci of all the strains were calculated using PAML package with the Nei-Gojobori method. Each figure represents the following: BB0120 (A), *wbmBB* (B), *wbmAA* (C), *wbmZ* (D), *wbmY* (E), *wbmX* (F), *wbmW* (G), *wbmV* (H), *wbmU* (I), *wbmT* (J), *wbmS* (K), *wbmR* (L), *wbmQ* (M), *wbmP* (N), *wbmO* (O), *wbmN* (P), *wbmM* (Q), *wbmL* (R), *wbmK* (S), *wbmJ* (T), *wbm*I (U), *wbmH* (V), *wbmG* (W), *wbmF* (X), *wbmE* (Y), *wbmD* (Z), *wbmC* (AA), *wbmB* (AB), *wbmA* (AC), and *wlbL* (AD). Click here for file

Additional file 3 **Evidence for site-specific positive selection in the O-antigen.** The posterior probability (P value) that individual codon positions belong to the positively selected category was calculated with PAML. Click here for file

Additional file 4 **Evidence for positive selection in *wbmC* and *wbmF* when taking into account recombination.** No evidence for positive selection in *wbmC* and *wbmF*, using PARRIS that takes into account recombination. Click here for file

Additional file 5 **GC content of the classical bordetellae strains.** The O1 type O-antigen loci (A), O2 type O-antigen loci (B), O- type O-antigen loci (C), or O3 type O-antigen loci (D) GC content of the classical bordetellae strains is plotted in 1,000 base pair increments. The overall average GC content of the entire genome is indicated by the red line, and the blue lines represent the standard deviation across the genome. Click here for file

Additional file 6 **Phylogenetic analysis of genes within the O-antigen locus.** Phylogenetic trees were constructed by maximum likelihood method with 1,000 bootstrap replicates with individual gene sequences in the locus. Each figure represents the following: *wbmBB* (A), *wbmAA* (B), *wbmZ* (C), *wbmU* (D), *wbmT* (E), *wbmS* (F), *wbmR* (G), *wbmQ* (H), *wbmP* (I), *wbmO* (J), *wbmN* (K), *wbmM* (L), *wbmL* (M), *wbmK* (N), *wbmJ* (O), *wbm*I (P), *wbmH* (Q), *wbmG* (R), *wbmF* (S), *wbmE* (T), *wbmD* (U), *wbmC* (V), *wbmB* (W), and *wbmA* (X). Click here for file

Additional file 7 **Effects of LPS vaccination on nasal cavity and trachea colonization of *B. bronchiseptica *strains RB50, 1289, and MO149.** C57/BL6 mice were vaccinated with purified LPS (100ng/per mouse) from RB50 (green), 1289 (red), MO149 (blue), or were sham vaccinated with PBS (white) on days 0 and 14. On day 28, mice were inoculated with 10^4^CFU of RB50, 1289 or MO149, and nasal cavity (A) and trachea (B) colonization were determined 3days post-inoculation. The error bars represent standard deviation of 4 mice per group. Click here for file
