# Supplementary figures and images for: Horizontally acquired divergent O-antigen contributes to escape from cross-immunity in the classical bordetellae
Source: BMC Evol Biol. 2013 Sep 25;13:209. doi: 10.1186/1471-2148-13-209 (PMC3849452; doi:10.1186/1471-2148-13-209)

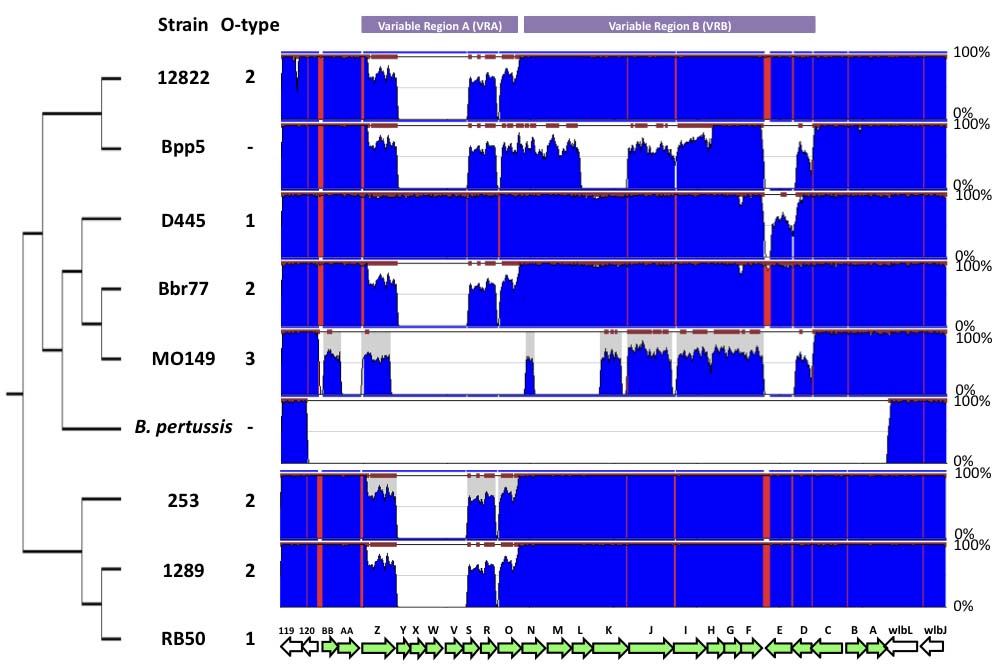

Supplement: Additional file 1 — Sequence similarity across the classical bordetellae O-antigen loci compared to B. bronchiseptica RB50. Percent similarity of the O-antigen locus and flanking genes based on RB50 was plotted between 0% and 100% using zPicture [42]. Intergenic regions and coding regions were highlighted with red and blue, respectively. The genome-wide SNP tree was superimposed on the left. [file 1471-2148-13-209-S1.jpeg]

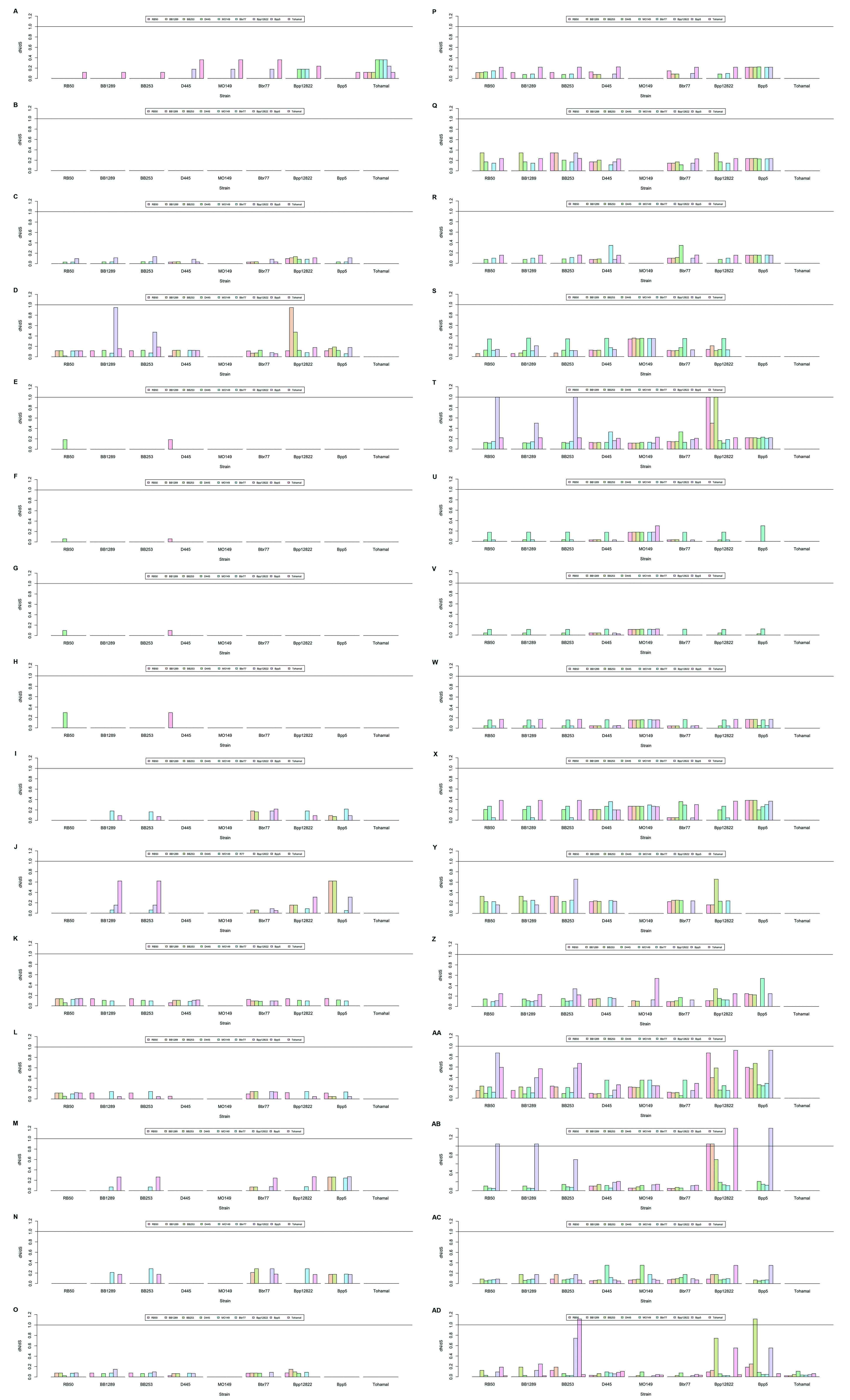

Supplement: Additional file 2 — Pair-wise dN/dS ratios of individual genes within the O-antigen loci. Pair-wise dN/dS ratios for the genes in the O-antigen loci of all the strains were calculated using PAML package with the Nei-Gojobori method. Each figure represents the following: BB0120 (A), wbmBB (B), wbmAA (C), wbmZ (D), wbmY (E), wbmX (F), wbmW (G), wbmV (H), wbmU (I), wbmT (J), wbmS (K), wbmR (L), wbmQ (M), wbmP (N), wbmO (O), wbmN (P), wbmM (Q), wbmL (R), wbmK (S), wbmJ (T), wbmI (U), wbmH (V), wbmG (W), wbmF (X), wbmE (Y), wbmD (Z), wbmC (AA), wbmB (AB), wbmA (AC), and wlbL (AD). [file 1471-2148-13-209-S2.jpeg]

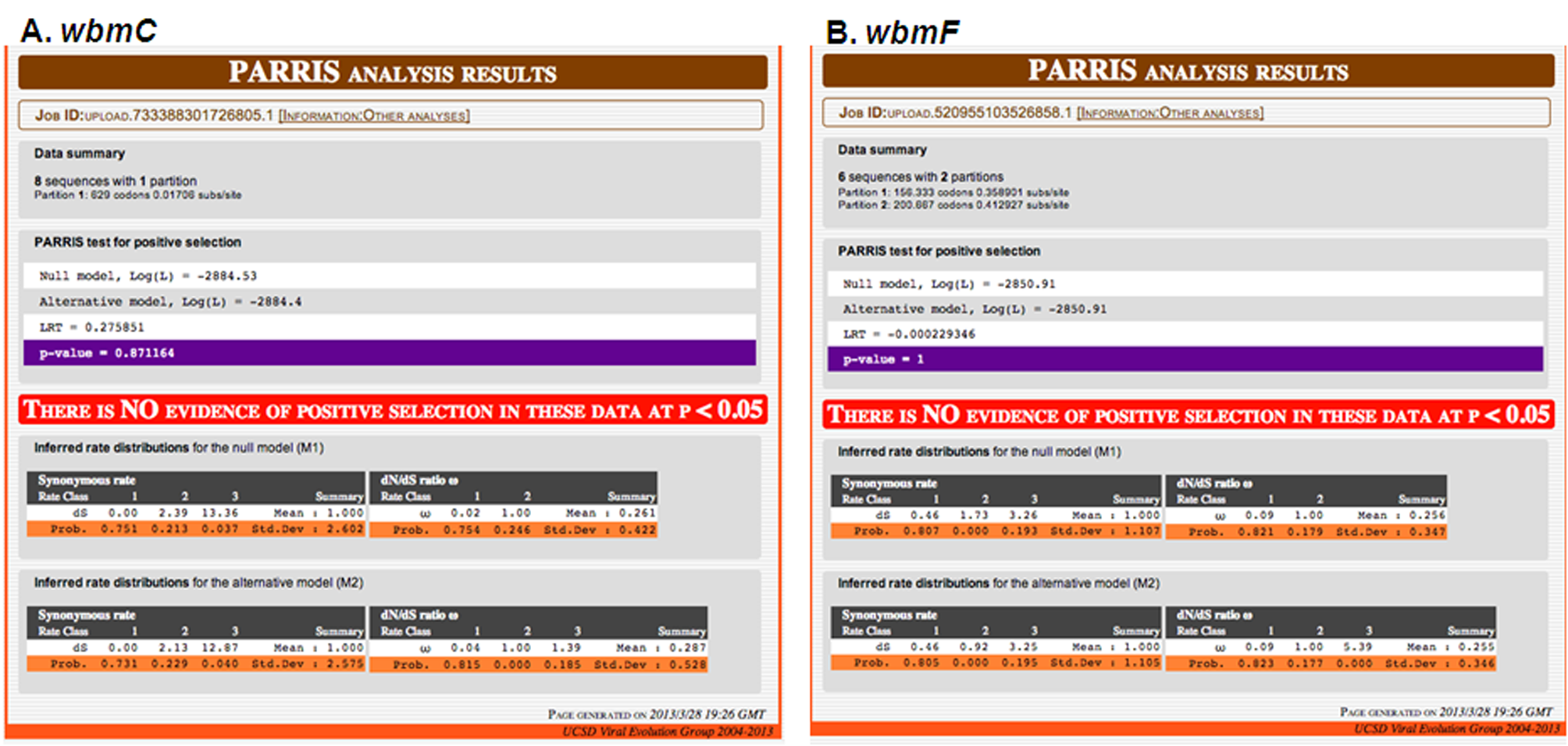

Supplement: Additional file 4 — Evidence for positive selection in wbmC and wbmF when taking into account recombination. No evidence for positive selection in wbmC and wbmF, using PARRIS that takes into account recombination. [file 1471-2148-13-209-S4.tiff]

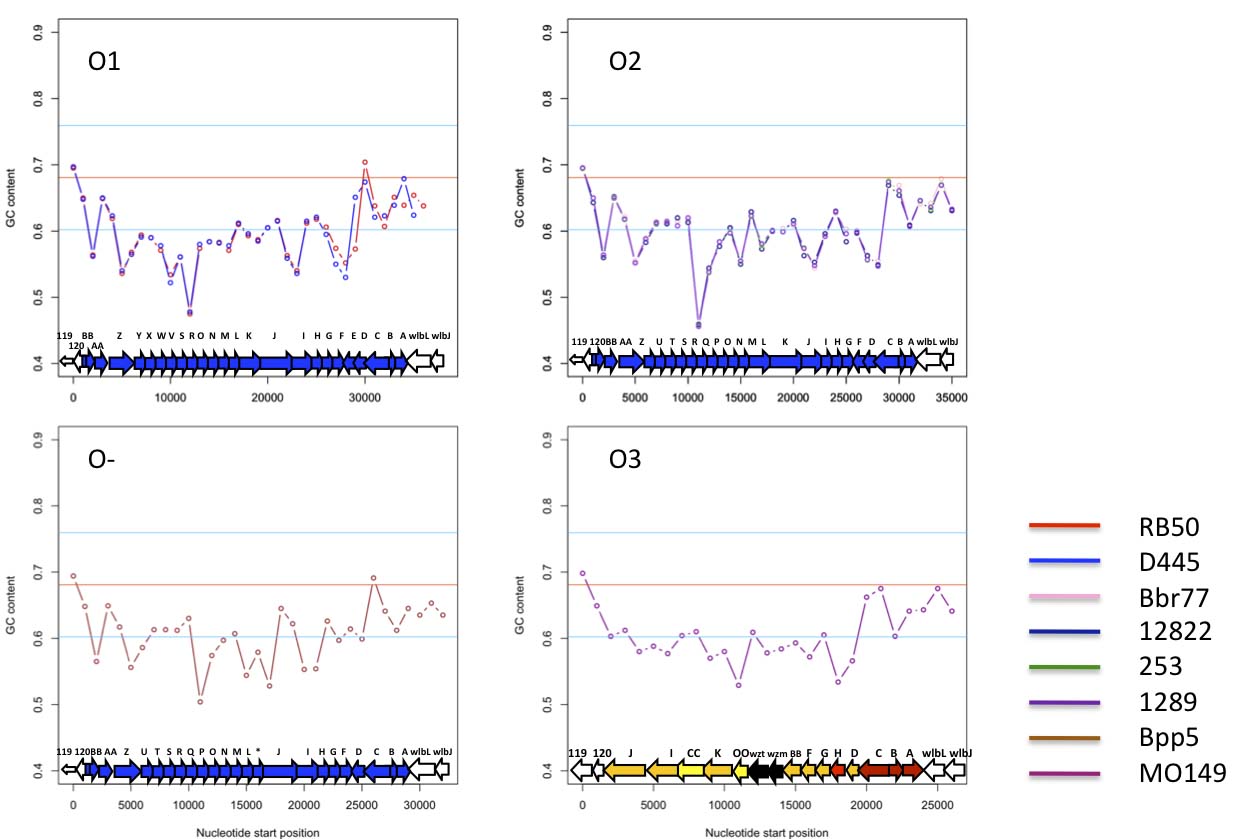

Supplement: Additional file 5 — GC content of the classical bordetellae strains. The O1 type O-antigen loci (A), O2 type O-antigen loci (B), O- type O-antigen loci (C), or O3 type O-antigen loci (D) GC content of the classical bordetellae strains is plotted in 1,000 base pair increments. The overall average GC content of the entire genome is indicated by the red line, and the blue lines represent the standard deviation across the genome. [file 1471-2148-13-209-S5.jpeg]

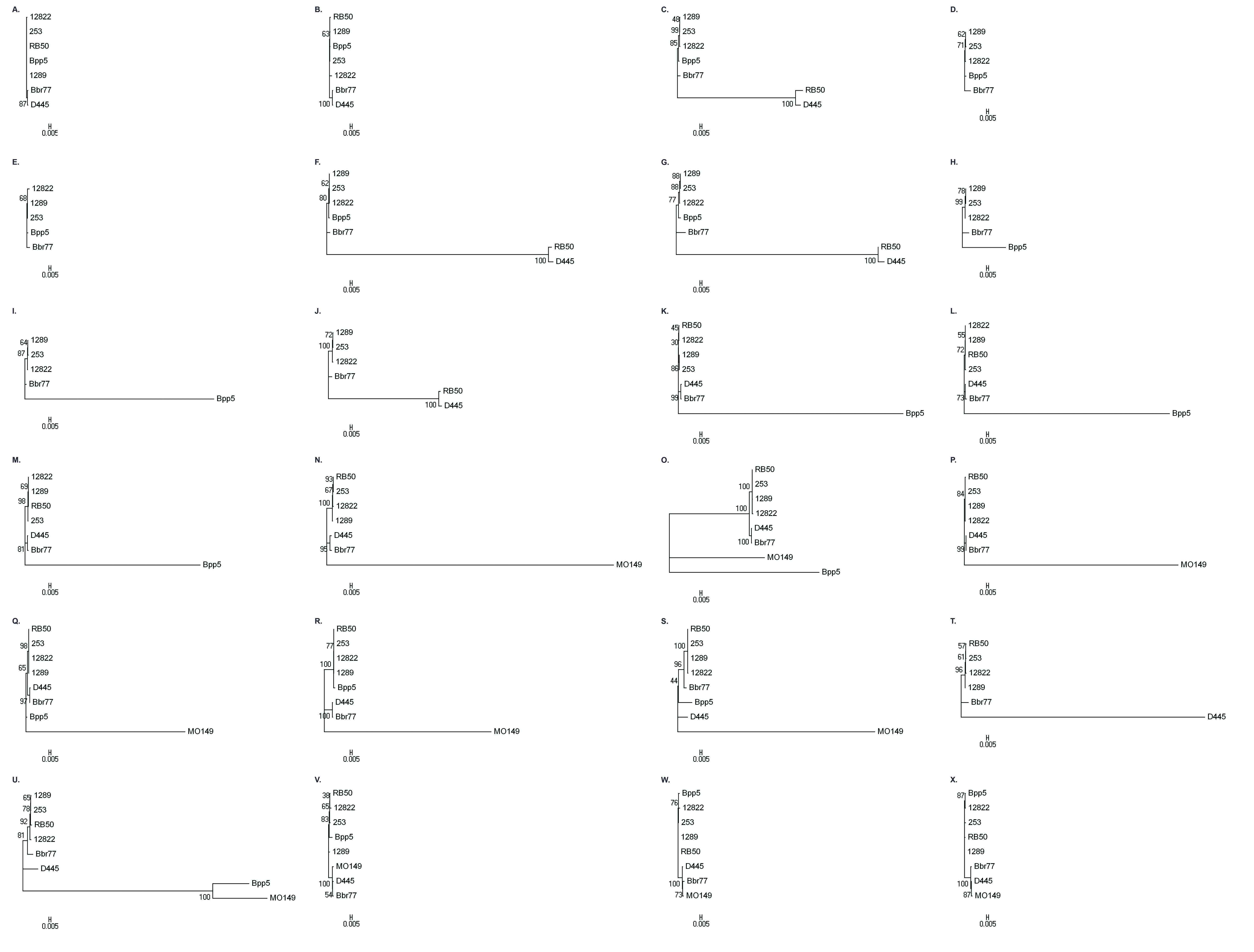

Supplement: Additional file 6 — Phylogenetic analysis of genes within the O-antigen locus. Phylogenetic trees were constructed by maximum likelihood method with 1,000 bootstrap replicates with individual gene sequences in the locus. Each figure represents the following: wbmBB (A), wbmAA (B), wbmZ (C), wbmU (D), wbmT (E), wbmS (F), wbmR (G), wbmQ (H), wbmP (I), wbmO (J), wbmN (K), wbmM (L), wbmL (M), wbmK (N), wbmJ (O), wbmI (P), wbmH (Q), wbmG (R), wbmF (S), wbmE (T), wbmD (U), wbmC (V), wbmB (W), and wbmA (X). [file 1471-2148-13-209-S6.jpeg]

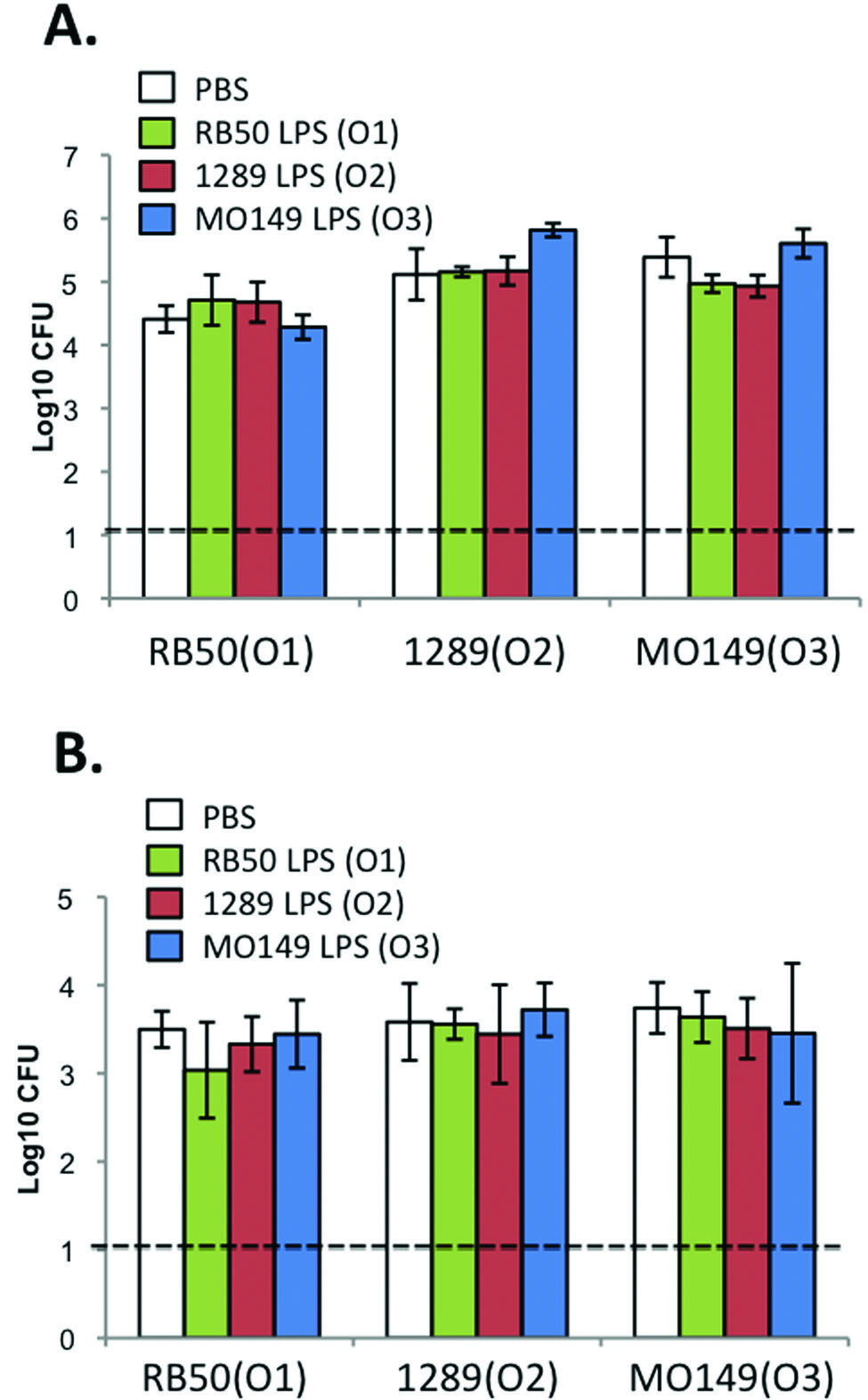

Supplement: Additional file 7 — Effects of LPS vaccination on nasal cavity and trachea colonization of B. bronchiseptica strains RB50, 1289, and MO149. C57/BL6 mice were vaccinated with purified LPS (100ng/per mouse) from RB50 (green), 1289 (red), MO149 (blue), or were sham vaccinated with PBS (white) on days 0 and 14. On day 28, mice were inoculated with 104CFU of RB50, 1289 or MO149, and nasal cavity (A) and trachea (B) colonization were determined 3days post-inoculation. The error bars represent standard deviation of 4 mice per group. [file 1471-2148-13-209-S7.jpeg]
